# Structures of Teneurin adhesion receptors reveal an ancient fold for cell-cell interaction

**DOI:** 10.1038/s41467-018-03460-0

**Published:** 2018-03-14

**Authors:** Verity A. Jackson, Dimphna H. Meijer, Maria Carrasquero, Laura S. van Bezouwen, Edward D. Lowe, Colin Kleanthous, Bert J. C. Janssen, Elena Seiradake

**Affiliations:** 1 Department of Biochemistry, Oxford University, OX1 3QU Oxford, UK; 2 Crystal and Structural Chemistry, Bijvoet Center for Biomolecular Research, Faculty of Science, Utrecht University, 3584 CH Utrecht, The Netherlands; 3 Cryo-electron Microscopy, Bijvoet Center for Biomolecular Research, Faculty of Science, Utrecht University, 3584 CH Utrecht, The Netherlands

## Abstract

Teneurins are ancient cell–cell adhesion receptors that are vital for brain development and synapse organisation. They originated in early metazoan evolution through a horizontal gene transfer event when a bacterial YD-repeat toxin fused to a eukaryotic receptor. We present X-ray crystallography and cryo-EM structures of two Teneurins, revealing a ~200 kDa extracellular super-fold in which eight sub-domains form an intricate structure centred on a spiralling YD-repeat shell. An alternatively spliced loop, which is implicated in homophilic Teneurin interaction and specificity, is exposed and thus poised for interaction. The N-terminal side of the shell is ‘plugged’ via a fibronectin-plug domain combination, which defines a new class of YD proteins. Unexpectedly, we find that these proteins are widespread amongst modern bacteria, suggesting early metazoan receptor evolution from a distinct class of proteins, which today includes both bacterial proteins and eukaryotic Teneurins.

## Abstract (web summary)

Teneurins are cell-cell adhesion receptors that evolved through horizontal gene transfer in which a bacterial YD-repeat protein fused to a eukaryotic receptor. Here the authors present crystallographic and cryo-EM structures of two Teneurins, revealing an ancient YD-repeat protein super-fold.

## Introduction

Teneurins (Ten1-4 in mammals) are functionally important type II single-pass transmembrane proteins^1–6^, which mediate adhesion between cells (in *trans*) through homophilic and heterophilic interactions of the extracellular region^1,2,7^. The intracellular domain of Teneurin is proteolytically cleaved and acts as a transcriptional repressor^8^. Homologue-specific Teneurin-mediated cell adhesion is crucial for neural wiring, e.g., in axon targeting and circuit formation of the visual system^6,9^ and in synaptic organisation^7^. Elegant recent experiments further reveal the importance of Teneurin in the wiring of the hippocampus, highlighting the importance of alternatively spliced loops that regulate homophilic Teneurin binding^10^. Teneurins have been linked with cancers^11^ and specific neurological disorders^12–14^. For example, a missense mutation in Ten1, P1610L, is associated with congenital general anosmia (the inability to smell), and mice carrying this mutation display an olfactory deficit^15^.

The Teneurin extracellular region defines its adhesive specificity and controls both homophilic and heterophilic *trans* interactions across the synapse. It contains eight membrane-proximal epidermal growth factor-like repeats (EGF1–8), which bear similarity to the vertebrate extracellular matrix protein tenascin^16–18^ and harbour intermolecular disulphide bonds that covalently dimerise Teneurins^19,20^. The EGF-like repeats are followed by a large 1850-residue segment of unknown structure, in which few parts have been annotated. A part of this segment has been derived from a bacterial tyrosine-aspartate (YD) repeat toxin by a horizontal gene transfer event during early metazoan evolution, an event that may have contributed to the evolution of multicellularity^21^. The YD-repeat motif has been structurally characterised in the context of heterodimeric bacterial toxin complexes (TcB-TcC), revealing that it forms a protective shell-like structure, encapsulating the proteolytically cleaved toxic C termini of these proteins^22,23^. In addition, the 1850-residue segment contains an ‘NCL-1, HT2A and Lin-41’ (NHL) domain that defines specificity for homophilic Teneurin *trans* interactions^24^.

Lack of detailed structural information on the Teneurin extracellular region has hampered progress with understanding Teneurin functions and related neurological diseases^15^. Here we present X-ray and electron microscopy (EM) structures for two out of four Teneurin (Ten2 and 3) ectodomains, revealing a ~200 kDa super-fold with a 170 kDa rigid core. Eight sub-domains, of which five were previously unreported, form an intricate structure centred on a spiraling YD-repeat shell domain. Unlike in known bacterial toxin structures of the YD-repeat family^22,23^, the ancient, now inactive C-terminal cytotoxic domain of Teneurin is included in our structure and, unexpectedly, lies outside of the shell. The shell is ‘plugged’ at the N-terminal side via a fibronectin (FN) and plug domain combination, which we also observed in current, structurally uncharacterized bacterial YD-repeat proteins. Taken together, our data provide structural insights into early receptor evolution, YD-repeat protein architecture and Teneurin function.

## Results

### Teneurins have a structurally conserved core fold

**Fig. 1:**
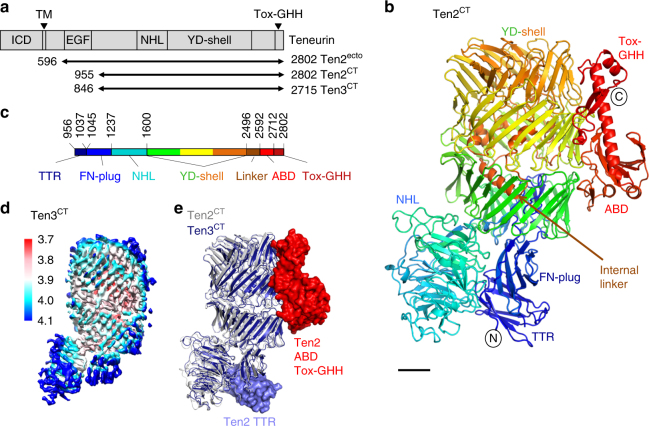
The Teneurin family super-fold. **a** Overview of Teneurin constructs used in biophysical and functional analysis. The predicted domain organisation of Teneurins based on the literature is shown alongside. **b** The 2.4 Å crystal structure of Ten2^CT^ is shown in ribbon representation and rainbow-coloured (blue, N terminus; red, C terminus). The position of each sub-domain is indicated. Scale bar = 1 nm. **c** The crystal structure was used to derive domain boundaries for each sub-domain in the Ten2^CT^ primary sequence. Colours are according to the model shown in **b**. TTR transthyretin-related, FN-plug fibronectin-plug; NHL NCL, HT2A and Lin-41, YD tyrosine-aspartate, ABD antibiotic-binding domain-like. **d** The 3.8 Å cryo-EM map of Ten3^CT^ is shown, coloured according to resolution. Colour key depicts resolution in angstrom. **e** The models of Ten2^CT^ (grey) and Ten3^CT^ (blue) are superposed and shown in ribbon representation. The TTR, ABD and Tox-GHH domains are disordered in the cryo-EM model of Ten3, and are shown in surface representation for Ten2^CT^ only

Here we reveal a conserved and surprisingly compact domain arrangement for the ~200 kDa C terminus of chicken Ten2 (Ten2^CT^, residues 955–2802, Fig. 1a–c) and mouse Ten3 (Ten3^CT^, residues 846–2715, Fig. 1a, c–e). The structures were solved by X-ray crystallography (Ten2^CT^, Supplementary Fig. 1) and single-particle cryo-EM (Ten3^CT^) to 2.4 and 3.8 Å resolution, respectively. This combination of structural techniques allowed us to assess the flexibility of Ten^CT^. Comparison of the two structures reveals a structurally conserved core fold with two flexibly attached domains (Fig. 1e). At their longest dimensions the structures measure 12.6 nm in length and 7.2 nm in width. The most N-terminal and most C-terminal segments are mobile and thus not resolved in the cryo-EM structure, resulting in a final refined Ten3^CT^ structure of residues 965–2512, which represents a rigid 170 kDa Teneurin core (Supplementary Figs 2 and 3). The two models are very similar (root mean squared deviation (r.m.s.d.) 1.4 Å for 1517 of 1525 aligned Cα atoms), consistent with the high level of sequence identity found for Teneurins (71% for chicken Ten2^CT^ and mouse Ten3^CT^). Indeed, the human Teneurin paralogues share 58–70% sequence identity, suggesting that the large extracellular Ten structure is similar for all four mammalian paralogues.

### An FNIII-plug domain combination seals the shell

Previously unpredicted, the structures reveal three β-stranded domains upstream of the Teneurin NHL domain. The most N-terminal domain, resolved in Ten2^CT^, is formed of seven β-strands arranged in two sheets, one antiparallel four-stranded and the other mixed three-stranded, both sandwiching a hydrophobic core. It bears structural similarity to transthyretin (TTR)-like domains and attaches peripherally onto the super-fold. TTR-like domains are widely distributed in species of bacteria, plants and animals, and gain their names from their structural similarity to TTR, a transport protein for thyroid hormones and retinol^25^. Other TTR-like domains have roles in protein aggregation^26^ and lipid recognition^27^.

**Fig. 2:**
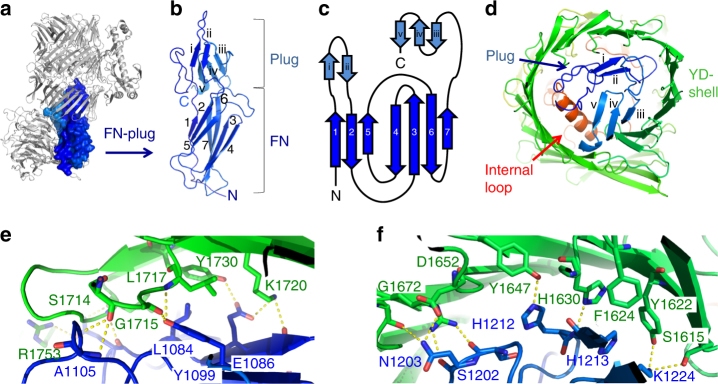
The FN-plug domain. **a** The Ten2 FN-plug domain is shown in surface representation and coloured as in Fig. 1b. The rest of the protein is shown as grey ribbons, oriented as in Fig. 1b. **b** The FN-plug domain (residues 1045–1237 in Ten2) is shown as ribbons. The FN and plug sub-domains are indicated. **c** The secondary structure of the FN-plug domain is shown as a cartoon where each arrow represents a β-strand. All β-strands are numbered as in **b**. **d** View of the FN-plug domain (blue) showing its position within the YD shell (green). The strands of the FN-plug domain are numbered as in **b**. **e**, **f** Zoomed views depicting selected interfacing residues of the FN-plug and YD-shell domains. A plot of all interfacing residues is provided in Supplementary Fig. 3

Downstream lies a FN type-III domain that contains a 44-amino-acid insertion between the first two β-strands and a 38-amino-acid extension at its C terminus (Fig. 2b, c). Together, these amino acids form a separate domain with no similarity to other known folds. We designate this the ‘plug’ domain, as it resides inside the shell cavity, acting to ‘seal’ its N-terminal side (Fig. 2a, d). Both FN and plug are also resolved in Ten3^CT^. The FN-plug domain forms numerous hydrogen-bonding and hydrophobic interactions with the shell interior (Fig. 2e, f and Supplementary Fig. 4).

### The NHL β-propeller is exposed

**Fig. 3:**
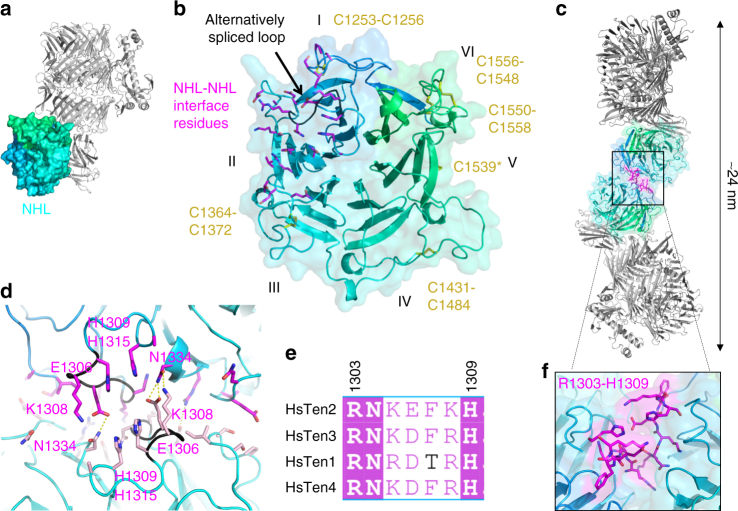
The NHL domain. **a** The Ten2 NHL domain is shown in surface representation and coloured as in Fig. 1b. The rest of the protein is shown as grey ribbons, oriented as in Fig. 1b. **b** The NHL domain viewed from the outward-facing side (top face). Individual blades of the propeller are labelled with roman numerals. Residues involved in an NHL-NHL crystal contact between symmetry-related Ten2^CT^ monomers are shown as sticks and coloured in magenta. The backbone of an alternatively spliced loop in the Ten2 NHL domain is coloured in black. **c** The symmetry-related monomers of Ten2^CT^ are shown. The view onto the loop central to this interface (R1303-H1309) is magnified in **f**. **d** Zoomed view depicting selected interfacing residues in the NHL-NHL contact. **e** Sequence alignment of the alternatively spliced residues located in the NHL-NHL interface. Numbers correspond to those for human Ten2. Hs *Homo sapiens*. **f** Zoomed view of the alternatively spliced residues (shown in magenta) in the NHL-NHL interface

Downstream of the FN-plug, the NHL domain forms a six-bladed β-propeller. It packs against the TTR, FN and shell domains with the bottom face (Figs. 1 and 3a). It is held in place by the FN domain, which acts as a wedge to position the NHL at an angle perpendicular to the YD-shell domain (Fig. 1b). The top face of the NHL propeller contains extended loops, stabilised by five disulphide bonds, all highly conserved amongst vertebrate Teneurins (Fig. 3b). Earlier studies showed that the NHL determines the specificity of homophilic Teneurin binding^24^, although the detailed structural signatures underlying this specificity have not been mapped. Indeed, the Ten2^CT^ NHL provides an extended crystal lattice contact between adjacent antiparallel monomers, burying ~1100 Å^2^ surface area (*S*_c_ = 0.46)^28^. The interface is formed by multiple hydrogen bonds and hydrophobic interactions (Fig. 3c, d). This may be representative of the Teneurin homophilic *trans* interaction surface (Fig. 3c–f). Key to forming this interface are residues of the alternatively spliced NHL loop (residues 1303–1309, Fig. 3e). Residues E1306 and K1308 of this alternatively spliced region form multiple hydrogen bonds across the interface (Fig. 3d). Consistent with these residues being important in homophilic Teneurin interactions in *trans*, previous reports including this sequence have shown cell–cell adhesive properties for Ten2^24^, whilst a report using the isoform that lacks these residues did not show adhesion for Ten2^5,29^. In addition to this, recent results indicate that inclusion of the corresponding splice site in Ten3 enables homophilic Teneurin interactions in *trans*^10^.

### A large 78-stranded YD shell at the centre of the structures

**Fig. 4:**
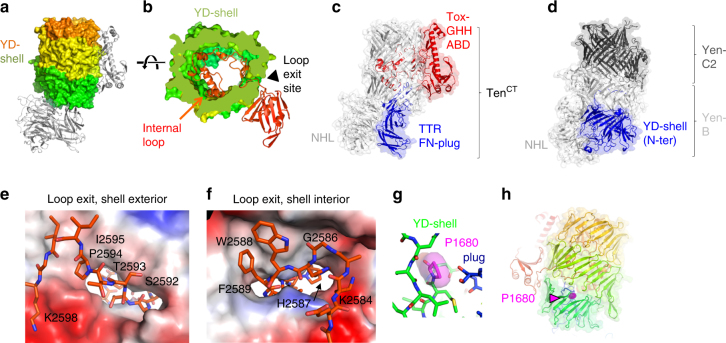
The YD shell and internal linker. **a** The Ten2 YD-shell domain is shown in surface representation and coloured as in Fig. 1b. The rest of the protein is shown as grey ribbons, oriented as in Fig. 1b. **b** A cut-through view of the YD shell (green) shown as a surface view. The internal loop and ABD (red) are shown as ribbons. The loop exit site on the shell is indicated. **c** Ten2^CT^ is shown in ribbons and transparent surface. The C-terminal domains not present in previously determined structures of bacterial toxin proteins^22,23^ are shown in red; the N-terminal FN-Plug and TTR domains, which lie upstream of the NHL domain, are in blue. **d** The structure of YenB/YenC2 is shown for comparison with Ten2 in **c**. Here the NHL domain is inserted into the YD-repeat domain. YD repeats that lie upstream of the NHL domain are shown in blue. **e**, **f** Zoomed views of the outside (**e**) and inside (**f**) surfaces of the YD shell at the loop exit site. The shell surface is coloured according to charge (negative = red, positive = blue, uncharged = white). The exiting loop is shown in red and as sticks. **g** Zoomed view of the conserved proline residue (P1680 in Ten2), mutated in Teneurin1-dependent anosmia, shown in magenta. Otherwise the colours are as in Fig. 1. **h** The position of the residue P1680 (shown as magenta spheres) is shown mapped onto the structure of Ten2^CT^. The rest of the model is shown as ribbons and coloured as in Fig. 1b

Downstream of the NHL lies a series of YD or rearrangement hotspot (Rhs) repeats, forming a striking ~800 residue YD-shell domain (Fig. 4a, b). It forms a 7.8 × 4.8 nm hollow tube, consisting of a single up-and-down spiralling β-sheet with 78 strands (Fig. 1b). The shell bears similarity to structures of bacterial toxin complexes, TcB-TcC^22,23^, although unlike these heterodimeric YD-repeat proteins, a single polypeptide chain forms the Teneurin shell. In the bacterial proteins the C-terminal end of the shell is closed by an Rhs-associated core domain (residues 2419–2496 in Ten2 and 2334–2411 in Ten3). This domain is the most structurally conserved region of Teneurins and the TcB-TcC toxin complexes (r.m.s.d. 1.48 Å for 66 of 81 aligned Cα atoms of the Rhs-associated core domain of *Yersinia entomophaga*, YenC2; Supplementary Fig. 5a). The auto-proteolytic motif found in the bacterial toxins (Supplementary Fig. 5b) is only partially conserved in Teneurins, which is consistent with our data showing that neither Ten2^CT^ nor Ten3^CT^ are cleaved at this position. Unlike Teneurins, which are N-terminally plugged by the FN-plug, TcB-TcC toxin complexes contain extended shell structures upstream of the NHL domain (Fig. 4c, d). Given these dissimilarities, we searched for bacterial sequences that encode Teneurin-like proteins, and that are therefore more likely to represent their evolutionary ancestors. Indeed, we found a series of bacterial protein sequences containing FN-plug, NHL and YD shell. We created a high-confidence homology model (GMQE 0.44, QMEAN −2.12^30^) for the protein with highest sequence identity to this region of chicken Ten2 (a protein from *Bacillus subtilis* (*B*. *subtilis*), NCBI accession WP_08111228.1, 27% identity; Supplementary Fig. 5c, d). Further sequence analysis demonstrates that Teneurin-like YD shells plugged by an FN-plug domain are widespread amongst Gram-positive and Gram-negative bacterial proteomes (Supplementary Fig. 6a, b), and represent a previously unknown ancient bacterial fold.

**A linker seals the YD shell and threads through it**. The Ten2^CT^ FN-plug and YD-shell combination encloses a volume of ~130 nm^3^. The residues downstream of the Rhs-associated core (residues 2467–2592 in Ten2^CT^ and 2382–2508 in Ten3^CT^) are located within the shell itself. This ‘internal linker domain’ is well resolved and has no detectable structural homologues (Fig. 4b). Its extended loop and helical elements act to seal small gaps in the wall of the YD shell, and also pack against the Rhs-associated core domain and FN-plug domain. Only 44 nm^3^ of internal shell space remains unoccupied by plug, Rhs-associated core domain and internal linker. In contrast to the TcB-TcC toxin complex family of proteins, where the C terminus of the C-domain resides inside the shell, a striking feature of the Teneurin structure is that the C terminus leaves the interior of the shell via a gap in the shell wall (Fig. 4e, f). Superposition with the published structure of a TcB/TcC protein from *Y. entomophaga*, YenB/C2^22^ shows that the gap is located in an area of the shell that corresponds to where the two subunits meet in the bacterial protein (Supplementary Fig. 7). The residues forming the gap are conserved across Teneurin paralogues.

The anosmia-causing mutation (P1610L in human Ten1) maps to a highly conserved area in the Ten2^CT^ shell (P1680, Fig. 4g, h and Supplementary Fig. 7). We found that P1680L mutant Ten2^ecto^ protein still binds to the canonical ligand Latrophilin (Supplementary Fig. 8a)^31^, suggesting it is folded properly and the disease is not likely due to impaired Latrophilin binding.

### Antibiotic-binding and Tox-GHH domains reside outside the YD shell

**Fig. 5:**
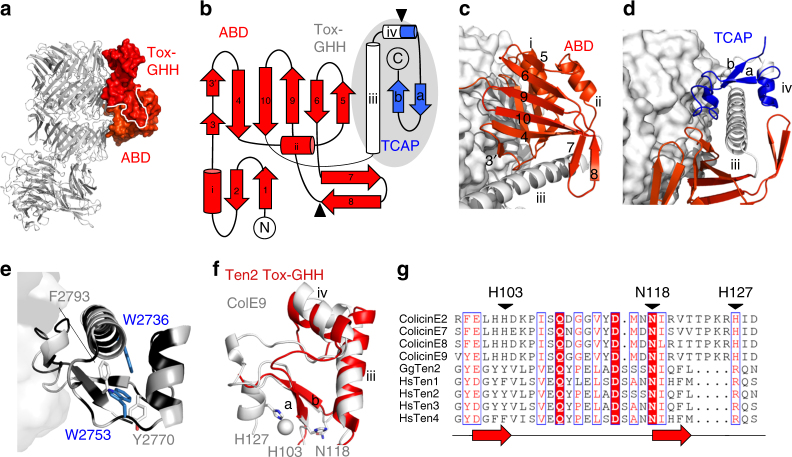
The ABD and Tox-GHH domain. **a** The Ten2 ABD and Tox-GHH domain are shown in surface representation and coloured as in Fig. 1b. The rest of the protein is shown as grey ribbons, oriented as in Fig. 1b. **b** Schematic of the ABD and Tox-GHH folds. The Tox-GHH area is indicated by a grey oval. Within the Tox-GHH, the TCAP area is shown in blue. Reported protease cleavage sites are indicated by arrowheads. **c** The ABD is shown as red ribbons. The shell surface is coloured white. The Tox-GHH domain is shown as white ribbons. **d** View of the Tox-GHH domain, coloured white except for the TCAP region, which is coloured in blue. The shell is shown as white surface, the ABD is shown as red ribbons. **e** View of the Tox-GHH domain as ribbons and the shell as surface. Residues are coloured according to the sequence conservation score of each residue (black = conserved, white = not conserved). Selected hydrophobic residues, all of which are conserved in Teneurins, are labelled and side chains shown as sticks (white from the TCAP area, blue from upstream Tox-GHH areas). **f** Overlay of the Ten2 Tox-GHH domain (red ribbon) and the nuclease domain of Colicin E9 (white ribbon, PDB 1BXI). Side chains of catalytically important residues are shown as sticks, and the catalytically important ion in Colicin E9 shown as a grey sphere. **g** Structure-based sequence alignment of the *Escherichia coli* DNase colicins, all four human Teneurin paralogues and chicken Ten2. The alignment corresponds to the most C-terminal two β-strands of the Ten2^CT^ crystal structure. The catalytically important residues of the Colicins are indicated by arrowheads. Each arrow corresponds to one β-strand

Downstream of the shell exit site, the structure reveals a small, 120-residue, domain (Fig. 5a, b) that bears structural homology to bacterial small-molecule antibiotic-binding domains (ABDs; Supplementary Fig. 5c,d). The ABD wraps around a long α-helix that leads into the very C-terminal region of Teneurin (residues 2712–2798 in Ten2^CT^, Fig. 5c, d). This was shown to have sequence similarity to the Tox-GHH family of DNAses^32^. In our Ten2^CT^ structure, the Tox-GHH domain reveals highest structural similarity to the catalytic domains of the colicin DNAse family of bacteriocins (*Escherichia* *coli* colicin E9: r.m.s.d. 2.65 Å for 60 aligned Cα atoms^33^; Fig. 5e, f), which is distinct from the TcB-TcC toxin complex family. Tox-GHH nucleases typically rely on a catalytic triad of two histidines and one asparagine (HNH), however these are mutated in Teneurins, rendering their nuclease activity inactive (Fig. 5g). Intriguingly, the vestigial HNH motif is located within the ‘Teneurin C-terminal-associated peptide’ (TCAP) region of the protein (Fig. 5g), which is predicted to be cleaved in vivo and act as a bioactive peptide. In vitro, this neuropeptide stimulates neurite outgrowth and controls dendritic morphology^34–38^. In vivo, TCAP is known to regulate anxiety behaviours of rats^38,39^. In our crystal structure, the TCAP is tightly associated with the YD shell and folds into a short N-terminal helix and a β-hairpin, connected by loops (Fig. 5d). The predicted proteolytic cleavage site, which is responsible for the release of TCAP from Teneurin in vivo^40^ lies within the second helix of Tox-GHH (Fig. 5b), suggesting that the protein may unfold in this area to become accessible to proteases. In support of this hypothesis, both the ABD and the Tox-GHH are mobile and not resolved in the cryo-EM structure of Ten3^CT^. In Ten2^CT^, the ABD is disordered in two of four molecules in the asymmetric unit.

## Discussion

Teneurins have recently emerged as vital specificity-determining cell–cell adhesion receptors^1–3^. Unlike previously described mammalian adhesion proteins, which contain small globular domain repeats in their extracellular regions, we show that Teneurins are derived from an evolutionarily ancient protein super-fold that is widespread across the bacterial kingdom. It appears in the genomes of both Gram-positive and Gram-negative species and is particularly prevalent amongst the proteobacteria. We show that a Teneurin-like protein of unknown function is encoded by *B*. *subtilis*. This protein harbours bacterial immunoglobulin-like (Ig) domains and a TTR-like domain upstream of its Teneurin-like core domain (Supplementary Fig. 5d). Ig domains are common in both bacterial and eukaryotic cell adhesion proteins, and this presents one possible function for the bacterial Teneurin-like proteins. Although the TTR-like domain found in the *B*. *subtilis* Teneurin-like protein shares relatively high sequence identity with that found in Teneurin (35% identity with the Ten2 TTR), an additional domain of unknown structure and function (~180 residues) separates the FN-plug and TTR in the *B*. *subtilis* protein. This domain of unknown function persists in the ancient Teneurins encoded by *Monosiga brevicollis* and *Branchiostoma sp*.^21^, but is absent in Teneurin proteins encoded by arthropods and higher chordates. Future analysis of this domain should provide further insights into the early evolution of Teneurins from bacterial proteins.

Amongst the bacterial Teneurin-like proteins, diverse uncharacterised C-terminal regions lie downstream of the YD repeats and Rhs-associated core domain, suggesting that the Teneurin-like core fold evolved as a platform or delivery machine to which diverse variable domains have been added. This is in agreement with previous reports suggesting that YD repeats and their associated C-terminal regions are evolutionarily decoupled in bacteria^41^. The Rhs-associated core domain of the bacterial Teneurin-like proteins shares higher sequence identity with that of TcC proteins than with the Teneurins, including sequence conservation of the auto-proteolytic cleavage site itself (which is not conserved in the Teneurins).

Homology modelling of the region downstream of the auto-proteolytic site reveals that some bacterial Teneurin-like proteins show similarity to lipases in this region, including conservation of catalytically important histidine residues (e.g., NCBI accession OGP50204.1). Together these observations suggest that the bacterial Teneurin-like proteins could be cleaved in their Rhs-associated core domain and could function as toxins.

The intricate nature of the fold begs the question, which functions are encoded in the conserved core of Teneurin and Teneurin-like proteins. The YD-shell of bacterial Teneurin-like proteins may act to hide a toxin that can be revealed upon a trigger (as postulated for the TcB-TcC toxins^22,23^), but has it merely been retained in Teneurins as a vast surface for protein–protein interaction? Suggestively, the gap in the YD-shell that forms the exit site for the C-terminal linker region in mammalian Teneurins coincides with the interface between the two separate subunits (B and C), of the TcB-TcC toxins. This prompts us to speculate that folding-intermediates, or indeed alternate functional states of Teneurin may exist, in which the shell is opened up at that position. Moreover, the Ten2^CT^ YD shell + FN-plug encloses a space of ~130 nm^3^. The C termini of the bacterial Teneurin-like proteins typically range from 100–150 amino acids in length, and are therefore not dissimilar to the length of the internal linker we report for Teneurin. This suggests that the C termini of the bacterial Teneurin-like proteins, some of which encode putative toxic activity, could be shielded within the YD shell, similar to what is observed for TcB-TcC toxins.

In multicellular animals, the addition of EGF domains to the Teneurin fold has added covalent *cis-*interaction functionality. Rotary shadowing EM data have revealed that these *cis* dimers are highly flexible^19^, presenting the large, globular Ten^CT^ structure on an elongated rod formed by the covalently dimerised EGF domains. Our own structural data support a model of NHL-NHL mediated *trans* interactions involving, at least in part, an alternatively spliced loop of the NHL domain. This is substantiated by recent results suggesting that inclusion of this alternatively spliced loop in Ten3 enables Ten3 homophilic interactions in *trans*^10^. However, at this stage we cannot exclude that NHL-NHL-mediated *cis* interactions involving this loop also play a role in Teneurin function, for example, homophilic *trans*-binding specificity could be modulated by the long-range structural effects of the NHL domains, even if those are involved in a *cis* interaction. Our data combined with previous reports^1,2,24^ now support a *trans*-synaptic Teneurin-mediated complex that may be tetrameric (a *trans*-dimer of *cis*-dimers) or array-like, reminiscent of assemblies postulated for other neuronal homophilic cell adhesion receptors, such as the protocadherins^42^. How heterophilic ligand interaction impacts on Teneurin structure and assembly-formation will need to be established.

## Methods

### Vectors and cloning

We cloned constructs of chicken Ten2^24^ (Q9DER5) (Ten2^CT^, residues 955–2802; Ten2^ecto^ residues 596–2802) and mouse Lphn2^Lec^ (Q8JZZ7, residues 30–137) into the AgeI-KpnI cloning sites of vectors from the pHLSec family^43^. Ten2^CT^ for crystallisation was fused to a C-terminal hexahistidine tag and Ten2^ecto^ (both wild type and P1680L) for SPR was fused to a C-terminal AviTag and hexahistidine tag. The plasmid encoding full-length mouse Ten3 lacks amino acids 741–749 (AHYLDKIVK) and amino acids 1219–1225 (RNKDFRH) (MGC premier cDNA clone BC145284, Biocat GmbH). Ten3^CT^ (residues 846–2715) was subcloned into the BamHI/NotI restriction sites of the pUPE107.03 vector (N-terminal cystatin secretion signal and C-terminal His6, U-Protein Express). Primers used for cloning and mutagenesis are shown in Supplementary Table 1.

### Protein expression and purification

We expressed all proteins in a secreted form using transiently transfected HEK293 cells^43^. Glycan maturation was inhibited during protein expression as specified below, to allow the structural analysis of homogenously glycosylated protein samples. Ten2 proteins were produced in kifunensine-treated HEK293T cells (American Type Culture Collection (ATCC) CRL-3216). Four days post transfection the cell culture medium was clarified by centrifugation and filtration and diafiltrated into 20 mM Tris (pH 7.5) and 500 mM NaCl. Ten3^CT^ was expressed in acetylglucosaminyltransferase I-deficient Epstein–Barr virus nuclear antigen I-expressing HEK293 cells (U-Protein Express, ATCC CRL-3022) and 6 days post transfection Ten3^CT^-containing medium was diafiltrated into 25 mM HEPES (pH 7.8) and 500 mM NaCl. All proteins were purified by Ni-NTA affinity chromatography and size-exclusion chromatography using an S200 16/60 column (Ten2 constructs) or a Superose6 column (Ten3 constructs, both GE Healthcare). For Ten2 constructs the gel filtration (GF) buffer was 20 mM Tris (pH 7.5) and 200 mM NaCl. The GF buffer for Ten3^CT^ was 20 mM CHES (pH 9) and 150 mM NaCl. Lphn2^Lec^ for SPR was produced in HEK293T cells without kifunensine. The medium was harvested 2 days post transfection and dialysed against phosphate-buffered saline (PBS) at 4 °C for 24 h.

### Protein crystallisation

Ten2^CT^ was purified as described above and concentrated to 5 mg ml^−1^ in 10 mM Tris (pH 7.5), 200 mM NaCl and 200 mM non-detergent sulfobetaine (NDSB-256). Initial, weakly diffracting crystals were grown using the vapour-diffusion method in sitting drops at 18 °C by mixing protein solution and crystallisation solution in a 1:1 ratio. The crystallisation solution was 0.1 M Tris (base)/Bicine (pH 8.5), 20% v/v glycerol, 10% w/v PEG4K, 30 mM di-ethyleneglycol, 30 mM tri-ethyleneglycol, 30 mM tetraethyleneglycol and 30 mM pentaethyleneglycol. Crystal quality was optimised by a combination of varying the drop ratio, linearly reducing the concentration of all components of the crystallisation solution and microseeding. Pt-derivative crystals were produced by adding K_2_PtCl_4_ in powder form to the drop containing Ten2^CT^ crystals and incubating for 21 h. Derivative crystals were back-soaked in crystallisation solution prior to flash-cooling. All crystals were cryo-cooled in the crystallisation solution described above.

### X-ray structure determination and refinement

Due to the radiation sensitivity of the crystals, 90° wedges of native diffraction data from three isomorphous crystals were collected up to 2.4 Å resolution on Diamond Light Source at 100 K (beamline I03, *λ* = 0.97626 Å). Wedges of native data having suffered the least radiation damage were selected for merging to produce the final native data set (Table 1). Data were indexed and integrated using XDS^44^, and scaled and merged using XSCALE (via Xia2)^45^.

**Table 1:** Data collection and refinement statistics for Ten2^CT^ data sets

	Pt-derivative	Native
PDB accession		6FB3
**Data collection**		
Space group	C222_1_	P2_1_
Cell dimensions		
* a*, *b*, *c* (Å)	83.32, 156.13, 452.95	88.56, 452.56, 146.36
* α*, *β*, *γ* (°)	90.00, 90.00, 90.00	90.00, 95.12, 90.00
Resolution (Å)	113.24–3.26 (3.34–3.26)^a^	90.51–2.38 (2.44–2.38)
* R*_meas_ (%)	34.3 (156.6)	25.0 (147.6)
* I*/σ*I*	7.17 (0.81)	4.69 (0.39)
CC_1/2_ (%)	99.3 (25.4)	99.0 (31.5)
Completeness (%)	99.9 (99.3)	96.3 (86.8)
Redundancy	12.2 (3.6)	5.6 (1.8)
**Refinement**		
Resolution (Å)	—	89.38–2.38
No. reflections	—	429 571 (25 518)
* R*_work_/*R*_free_	—	0.225/0.242 (0.307/0.319)
No. of atoms	—	
Protein	—	58 012
Ligand/ion	—	1216
Water	—	183
Average *B* factors (Å^2^)	—	76.85
R.m.s. deviations	—	
Bond lengths (Å)	—	0.007
Bond angles (°)	—	0.99
Validation	—	
MolProbity score	—	1.53
Clashscore	—	2.32
Poor rotamers (%)	—	2.28
Ramachandran Plot	—	
Favoured (%)	—	96.36
Allowed (%)	—	3.33
Disallowed (%)	—	0.37

The Pt-derivative data set was calculated by merging data from two crystals. The native data set combines data collected from three crystals

^a^Values in parentheses are for highest-resolution shell

For anomalous data collection, data sets from two isomorphous crystals were collected at the European Synchrotron Radiation Facility (beamline ID29) at 100 K. To maximise anomalous signal, data were collected at the L-III Pt edge (*λ* = 1.072 Å). Data were processed as above, but Bijvoet pairs were unmerged (Table 1). Crank2^46,47^ was used to calculate phases and build an initial model. The model was manually improved using Coot^48^ and the NHL domain was placed by molecular replacement in Phaser using a modifed polyalanine model of the β-propeller domain of a distant structural homologue (Protein Data Bank (PDB) accession 3HRP). The resulting model was subsequently used for iterative phase improvement using the Phaser SAD pipeline^49^ and manual re-building in Coot^48^. Partial sequence assignment was facilitated by homology models of the Ten2 Rhs-associated core domain and YD-shell region based on the structure of YenC2 (PDB 4IGL)^22^ and the knowledge that Pt atoms typically bind to either methionine or histidine residues.

The resulting structure was used to phase the higher-resolution native data by molecular replacement with Phaser^50^ to place four copies of the model in the P2_1_ asymmetric unit. The resulting map was density-modified using Parrot^51^ and then supplied to Buccaneer^52^ for autobuilding, allowing reliable assignment of much of the sequence. The remainder of the sequence was assigned manually based on the density. Glycans were built using the automatic glycan building implementation in Coot^48^. The model was all-atom refined in autoBUSTER using non-crystallographic symmetry (NCS) restraints^53,54^. Thermal motion was parameterized by treating each macromolecular chain as a single translation libration screw group. The quality of the final model was assessed using MolProbity^55^.

### Cryo-EM sample preparation

Purified Ten3^CT^ in GF buffer was diluted in MilliQ water to a final salt concentration of 75 mM NaCl and a final protein concentration of 100 μg ml^−1^. Three-microlitre aliquots of Ten3^CT^ were applied onto glow-discharged holey carbon Quantifoil R2/2 grids. Grids were blotted for 2 s at 95% humidity with a blot force of −2 using a Vitrobot Mark IV (FEI). Grids were stored in liquid nitrogen until further use.

### Cryo-EM data collection

The Ten3^CT^ data set was collected on a Talos Artica (FEI) operating at 200 kV, equipped with a GIF quantum energy filter (Gatan) and K2 Summit camera (Gatan). Movies were collected in super-resolution counting mode at a nominal magnification of 130 000, corresponding to a physical pixel size of 1.03 Å at the specimen level. The exposure time was 7.2 s, every 0.4 s a frame, corresponding with an electron dose of 56 e^−^ Å^−2^ for the integrated image and 3.1 e^−^ Å^−2^ per frame. A total of 2775 micrographs were collected using EPU software (FEI), with a defocus range of −1.2 to −3.0 µm.

### Ten3^CT^ Cryo-EM image processing

All micrographs were corrected for beam-induced motion and drift using MotionCor2^56^. After CTF modelling with Gctf^57^, micrographs with resolution estimates to 5 Å or better at CC = 50% were selected (see also Supplementary Fig. 2). A set of ~1000 particles were picked manually to generate four two-dimensional (2D) classes for automated particle picking in Relion2.0^58^. Particles were picked with a mask size of 200 Å in diameter, and a box size of 396 Å was used for particle extraction. After 2D classification, ab initio three-dimensional classification was performed in cryoSPARC^59^. CryoSPARC determines a reference free ab initio model using the Stochastic Gradient Descent algorithm. The best-looking class was refined using default dynamic mask settings (dynamic mask start at 12 Å resolution, with a soft-edge extending from 6 to 14 Å). The particle coordinates were further improved by particle polishing in Relion2.0 and local CTF determination in gCTF and imported back into cryoSPARC for a final refinement. The two resulting half maps were imported to Relion2.0 to calculate the Fourier Shell Correlation (FSC) by generating a mask that was extended by 2 Å and by adding a cosine-shaped soft edge of 4 Å. This resulted in a resolution of 3.8 Å at the FSC = 0.143 resolution criterion. Local resolution estimates were determined by the local resolution algorithm in Relion2.0 that samples the map with a soft-spherical mask.

### Cryo-EM model refinement

Using the structure of Ten2^CT^ as a template a model for Ten3^CT^ was generated in Modeller v9.16^60^. The TTR domain (residues 872–964), the ABD and Tox-GHH domains (residues 2513–2715) and residues 1219–1228, 1401–1411, 2413–2424 and 2487–2496 were omitted from the model due to lack of density in the Ten3^CT^ density map. The structure was refined by manual re-building in Coot^48^ and by real-space refinement in Phenix^61^ (Table 2). The missing N- and C-terminal segments were added to the refined structure Ten3^CT^. The model was validated using MolProbity^55^. The correlation between map and model was calculated in EMAN2 (Supplementary Fig. 2d). First, a map of the Teneurin-3 structure was generated at Nyquist using e2pdb2mrc.py and then the FSC was calculated using e2proc3d–calcfsc.

**Table 2:** Cryo-EM data collection and refinement statistics

	Ten3^CT^ (EMDB-4219) (PDB 6FAY)
Data collection and processing	
Magnification	130 000
Voltage (kV)	200
Electron exposure (e^–^ Å^−2^)	56
Defocus range (μm)	−1.2 to −3.0
Pixel size (Å)	1.03
Symmetry imposed	no
Initial particle images (no.)	435 755
Final particle images (no.)	287 402
Map resolution (Å)	3.8
FSC threshold	0.143
Map resolution range (Å)	3.7–4.1
Refinement	
Initial model used (PDB code)	Ten2^CT^
Model resolution (Å)	3.8
FSC threshold	0.3
Map sharpening *B*factor (Å^2^)	0
Model composition	
Non-hydrogen atoms	12 118
Protein residues	1525
Ligands	5
* B* factors (Å^2^)	
Protein	128
Ligand	117
R.m.s. deviations	
Bond lengths (Å)	0.005
Bond angles (°)	1.23
Validation	
MolProbity score	2.03
Clashscore	9.33
Poor rotamers (%)	0.75
Ramachandran plot	
Favoured (%)	90.63
Allowed (%)	9.24
Disallowed (%)	0.13

### Surface plasmon resonance

Equilibrium binding experiments were performed at 25 °C using a Biacore T200 instrument (GE Healthcare) using PBS + 0.005% (v/v) polysorbate 20 (pH 7.5) as running buffer. The regeneration buffer was 2 M MgCl_2_. Mouse Lphn2^Lec^ was biotinylated enzymatically in vivo at a C-terminal AviTag and coupled to a streptavidin-coated CM5 chip. Data were analysed using the BIAevaluation software. *K*_D_ and *R*_max_ values were obtained by nonlinear curve fitting of a 1:1 Langmuir interaction model (bound = *R*_max_/(*K*_D_ + *C*), where *C* is analyte concentration calculated as monomer).

### Data availability

Data supporting the findings of this manuscript are available from the corresponding authors upon reasonable request. Atomic coordinates have been deposited in the PDB with accession numbers 6FB3 (Ten2^CT^) and 6FAY (Ten3^CT^). The cryo-EM density map for Ten3^CT^ has been deposited in the Electron Microscopy Data Bank under the accession number EMD-4219.

## Electronic supplementary material

Supplementary Information
