# Supplementary material for: Structures of Teneurin adhesion receptors reveal an ancient fold for cell-cell interaction
Source: Nat Commun. 2018 Mar 14;9:1079. doi: 10.1038/s41467-018-03460-0 (PMC5851990; doi:10.1038/s41467-018-03460-0)
Supplement: Supplementary file 1 — Supplementary Information [file 41467_2018_3460_MOESM1_ESM.pdf]

Structures of Teneurin adhesion receptors reveal an ancient fold for cell-cell interaction

Supplementary Information

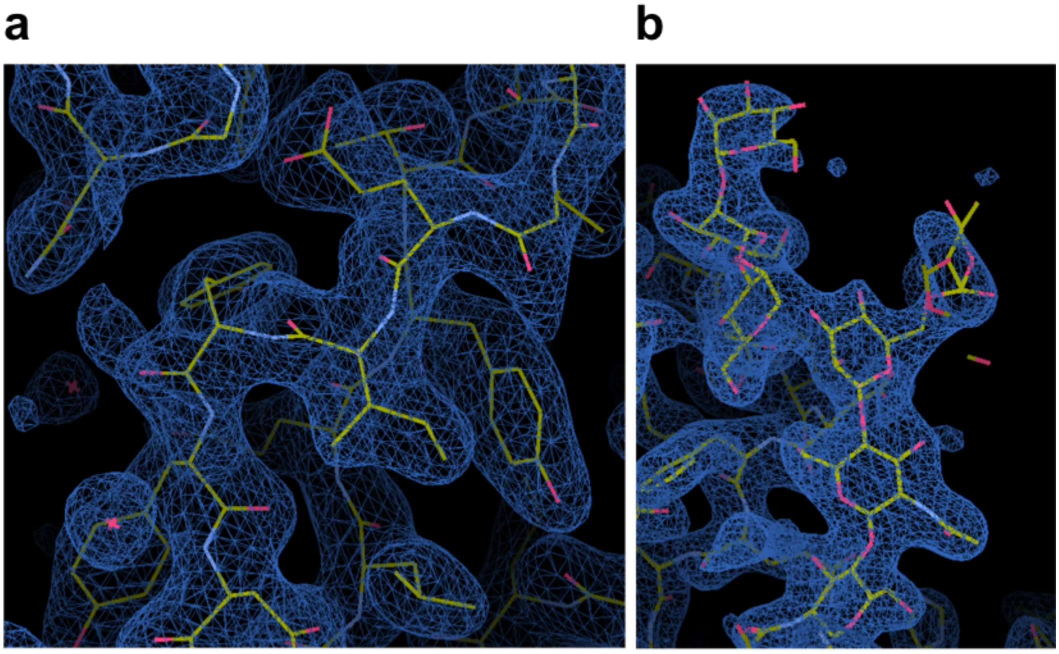

Supplementary Figure 1 | **Ten2^{CT} electron density map.** Final electron density (2Fo-Fc, contour 1.5σ) for **a,** protein and **b,** carbohydrate components of Ten2^{CT}. Atoms are coloured yellow (carbon), blue (nitogen) and pink (oxygen).

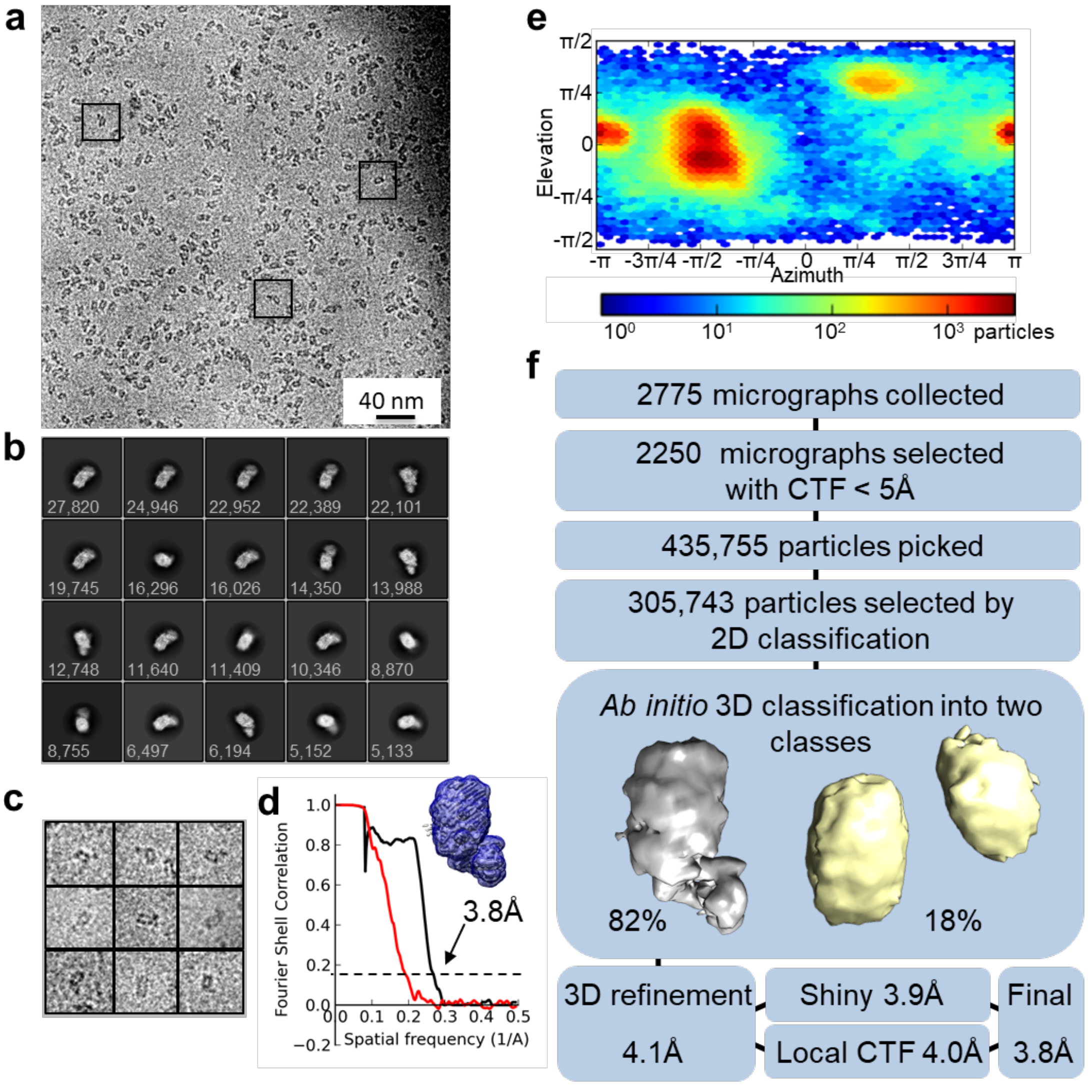

Supplementary Figure 2 | **Ten3^{CT} cryo-EM reconstruction a**, Selected micrograph at a defocus level of -3µm showing distribution of the particles. **b,** 2D classification without alignment of final particle set into 20 classes, generated with Relion. Number in gray represents number of particles in each class. **c,** A selection of raw particles from the final particles set. **d**, Fourier Shell Correlation (FSC) curve of two-independent half-maps of the 3.8 Å reconstruction. Dashed line indicates FSC=0.143. Phase-randomized FSC in red, corrected FSC in black. Inset shows mask used to generate FSC curve. **e**, The orientation distribution of the particles in the 3.8 Å reconstruction. **f**, Overview of the workflow for the 3.8 Å reconstruction using a variety of software packages as described in more detail in the Methods section.

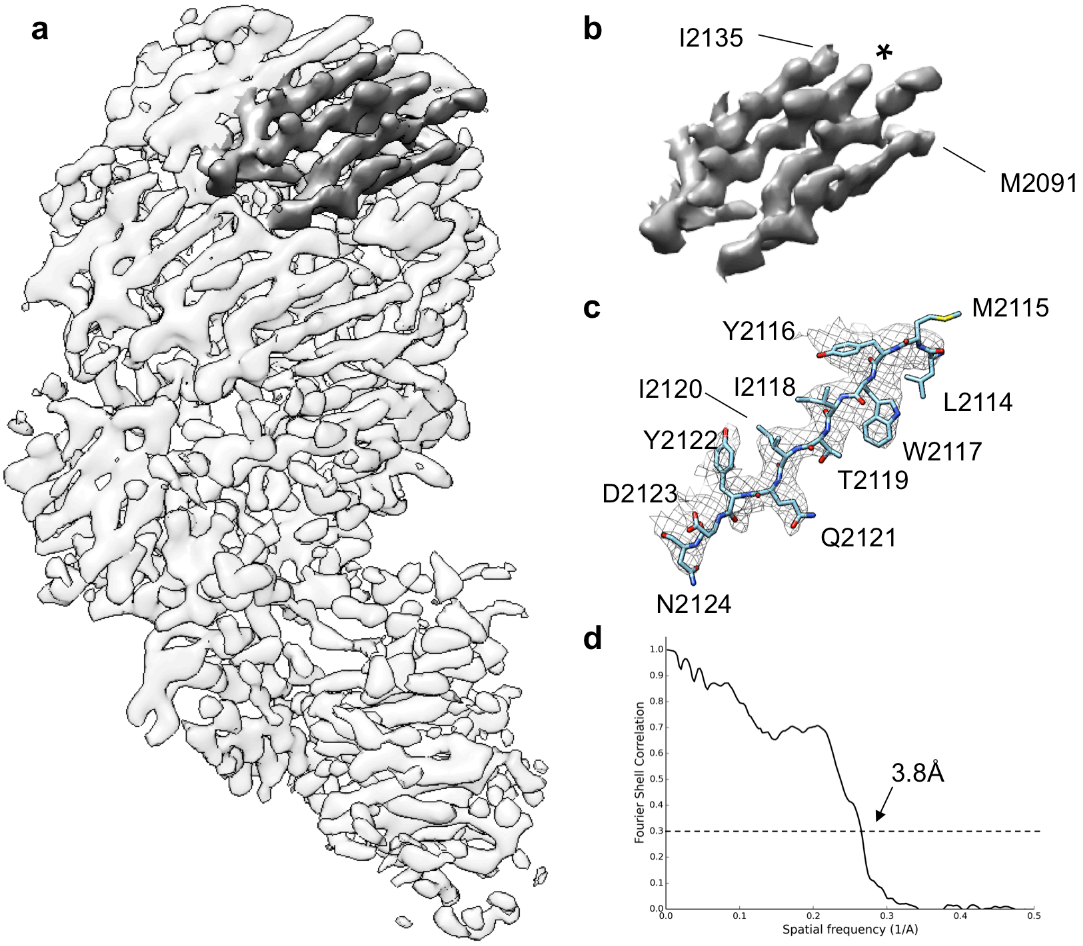

Supplementary Figure 3 | **Ten3^{CT} cryo-EM model building. a,** Side view of Ten3^{CT} map after B-factor sharpening with a B-factor of -100 Å^2, filtered by local resolution and HideDust setting 5. **b** β-strands are well separated at 3.8 Å. Shown here are residues 2091-2135. Asterisk indicates β-strand shown in panel **c**. **c**, Overlay of β-strand with Ten3^{CT} refined model spanning residues M2115 to N2124. **d**, Fourier shell correlation curve of Ten3^{CT} map versus Ten3^{CT} model. Dashed line indicates FSC=0.3.

YD Shell and FN-plug interfacing residues

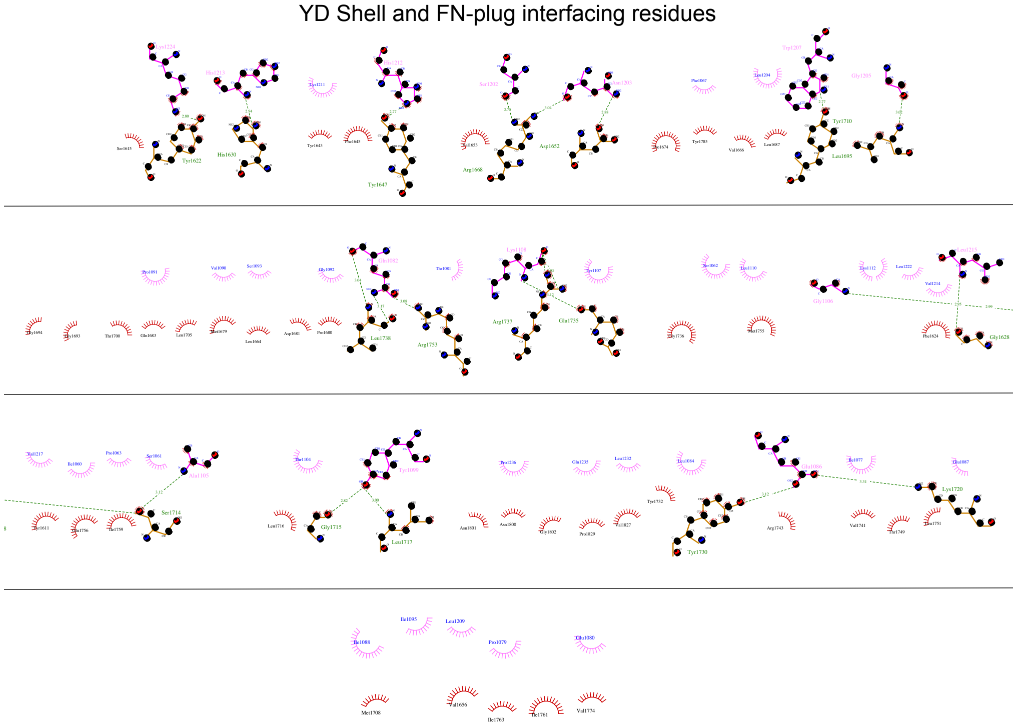

Supplementary Figure 4 | **The FN-plug and YD-shell interface**.

Interacting residues of Ten2 FN-plug (pink) and YD-shell domains (orange) were analysed and plotted using LigPlot(+) [1]. Hydrogen bonds are indicated for residues shown as sticks. Residues providing hydrophobic contacts are shown as arches. The linear representation of the interface is continued in sequential fashion, i.e. line-by-line and left to right.

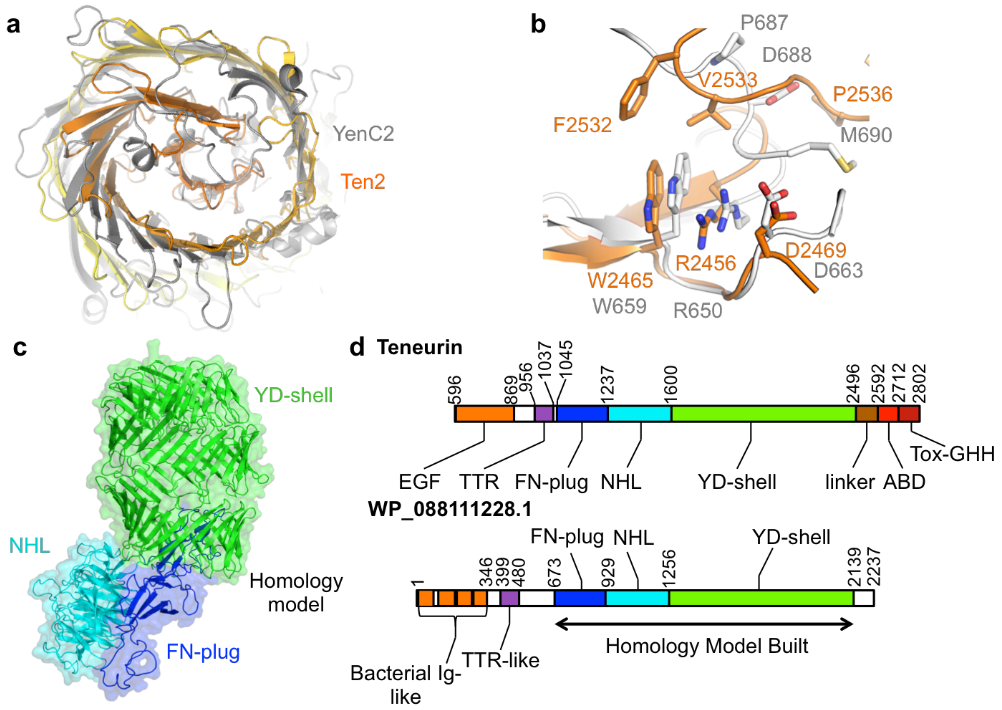

Supplementary Figure 5 | **Bacterial and Teneurin YD proteins. a.** Top view of the bacterial toxin YenC2 (grey) and the Ten2 (yellow to orange as in Fig. 1b) YD-shells aligned via their Rhs-associated core regions. **b.** Zoomed view of the auto-proteolytic site in the YenC2 Rhs-associated core region (white). Structural alignment of the Ten2 (orange) Rhs-associated core region shows conservation of some catalytically important residues (D663 and R650 in YenC2, corresponding to D2469 and R2456 in Ten2), but a substantial shift in the backbone for a region containing a third essential catalytic residue (D688 in YenC2). Side chains are shown as sticks. **c.** A high confidence homology model, based on the crystal structure of Ten2^{CT}, was produced by SWISS-MODEL (GMQE 0.44, QMEAN -2.12) from the *B. subtilis* protein WP_088111228.1. The results show it contains a Teneurin-like FN-plug, NHL and YD-shell. **d.** Schematics comparing the domain organization of Teneurin (upper panel) and *B. subtilis* protein WP088111228.1 (lower panel).

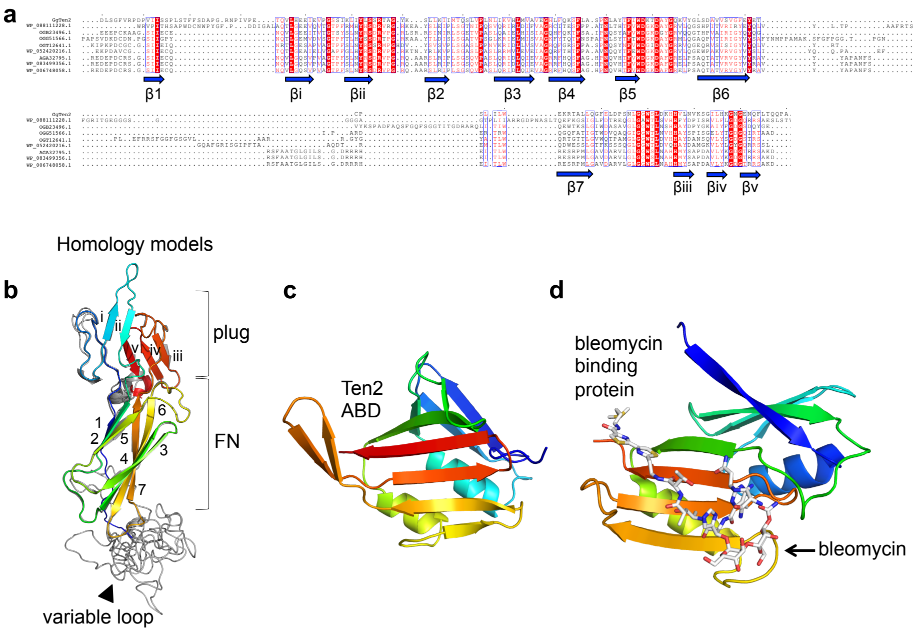

Supplementary Figure 6 | **Unpredicted prokaryotic-type domains found in the Teneurin super-fold. a**. Structure-based sequence alignment of the chicken Ten2 FN-plug domain with FN-plug domains from both gram positive and gram negative bacterial species. The secondary structure of the Ten2 FN-plug is shown below the alignment where each arrow represents a β-strand. β-strands are numbered as in Fig. 2b. **b.** Overlay of the Ten2 FN-plug domain (coloured according to the rainbow) and high confidence homology models of the bacterial FN-plug domains shown in **a** are coloured in grey. β-strands are numbered as in Fig. 2b. A variable loop between β-strands 6 and 7 is indicated. **c,d.** The Ten2 ABD and bleomycin binding protein (PDB 1EWJ) [2] are shown in ribbon representation and coloured according to the rainbow (blue, N-terminus; red, C-terminus). Bleomycin is shown as sticks. The equivalent bleomycin-binding site in the Ten2 ABD is solvent exposed in the Ten2^{CT} crystal structure.

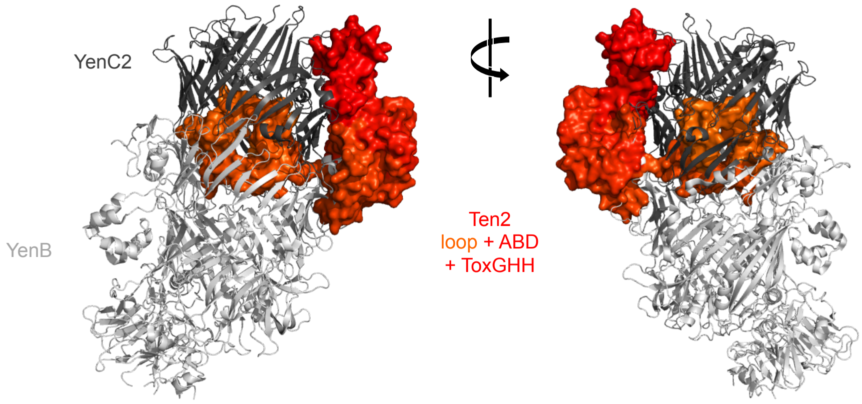

Supplementary Figure 7 | **Superposition of Ten2^{CT} and a bacterial TcB-TcC toxin.**
The bacterial YenB/C2 complex was structurally aligned with Ten2^{CT} via the C2 subunit, and is displayed as ribbons. B is in light grey, C2 is in dark grey. The Ten2 internal loop, ABD and Tox-GHH domains are depicted as surfaces, coloured as in Fig. 1b, highlighting where these domains and the internal loop exit site lie with respect to the bacterial proteins.

a

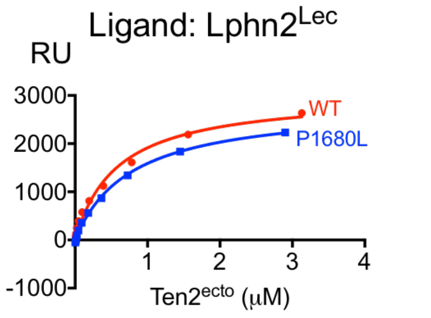

	K_D (μM)	R_{max} (RU)
WT	0.79 ± 0.11	3090 ± 140
P1680L	0.74 ± 0.07	2802 ± 82

b

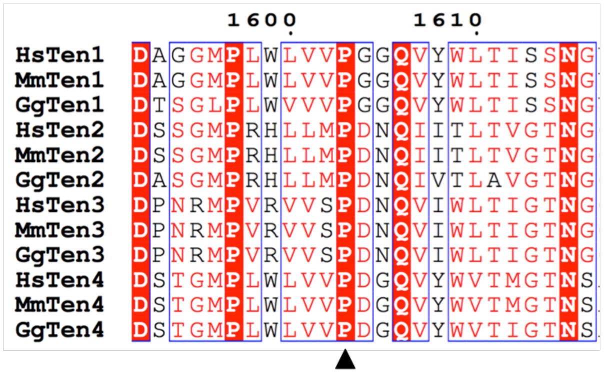

c

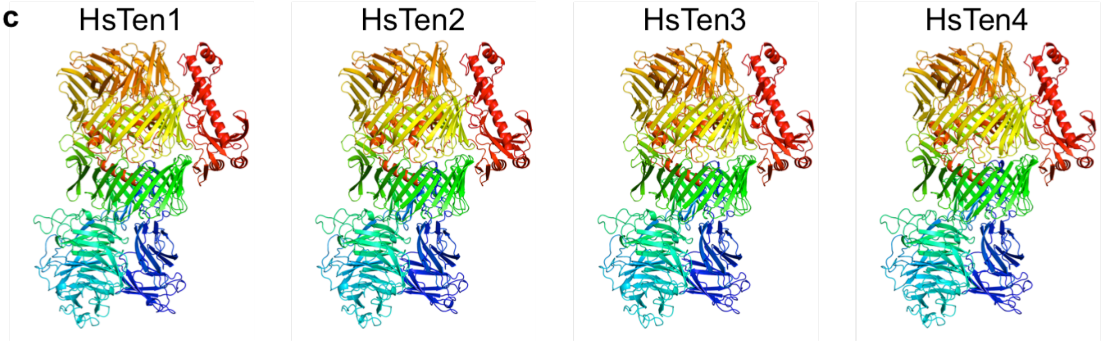

Supplementary Figure 8 | **Biophysical characterization of the Ten2 anosmia-linked P1680L mutation. a.** Lphn2^{Lec} was immobilized on the surface of a streptavidin-coated CM5 chip and different concentrations of Ten2^{ecto} (wild type and mutant, P1680L) injected as analytes. The data were fitted using a 1:1 binding model. The standard error of the K_D and R_{max} values is shown, demonstrating that the binding of Lphn2^{Lec} to Ten2^{ecto} is unaffected by the anosmia-linked mutation, P1680L. **b.** Sequence alignment showing 100% conservation of the proline residue mutated in congenital anosmia (marked with an arrowhead) across all Teneurin paralogues in human, mouse and chicken. **c.** High confidence homology models of all four human Teneurin paralogues based on the crystal structure of Ten2^{CT} demonstrating the high level of structural conservation across the family.

Supplementary Table 1

Cloning Primers			
Construct	Direction	Residue #	Sequence
Ten2^{CT}	Forward	955	GTAGCTGAAACCGGTAGCCTTGTGTCTCTTATAAGAGGCCAAGTG
Ten2^{CT}	Reverse	2802	GTGGTGCTTGGTACCCCTCTTTCCCATTTCATTCTGTCTTAAAAACTGG
Ten3^{CT}	Forward	846	AATAATGGATCCTTCTATGACCGAATCAGTTTCC
Ten3^{CT}	Reverse	2715	AATAATGCGGCCGCCCTCTTGCCGATCTCACTTTG
Ten2^{ecto}	Forward	596	GTAGCTGAAACCGGTGTTATTTTGGATTCAGTGCAAGACTGTCCACG
Ten2^{ecto}	Reverse	280	GGAACCTCCGGTACCCCTCTTTCCCATTTCATTCTGTCTTAAAAACTGG
Lphn2^{Lec}	Forward	30	GTAGCTGAAACCGGTGCCTTACCATTCGGGTTAGTTAGACGA
Lphn2^{Lec}	Reverse	137	GGAACCTCCGGTACCCACCAGCATCTTGCTGTAAGGGACACATTCATATTGAACTTCAAGG
**Mutagenesis Primers**			
Construct	Direction		Sequence
Ten2^{P1680L}	Forward		GATAATCAGATTGTCACGCTGGCCGTT
Ten2^{P1680L}	Reverse		GACAATCTGATTATCAAGCATCAGTAAATGGCGGGGCAT

Supplementary Table 1 | **Cloning and mutagenesis primers for Teneurin and Latrophilin constructs.**

Supplementary References

1. Laskowski, R. A. & Swindells, M. B. LigPlot+: Multiple ligand-protein interaction diagrams for drug discovery. *J. Chem. Inf. Model.* **51,** 2778–2786 (2011).
2. Maruyama, M. *et al.* Crystal structures of the transposon Tn5-carried bleomycin resistance determinant uncomplexed and complexed with bleomycin. *J. Biol. Chem.* **276,** 9992–9999 (2001).
